# A Validated Ultrasound-Assisted Extraction Coupled with SPE-HPLC-DAD for the Determination of Flavonoids in By-Products of Plant Origin: An Application Study for the Valorization of the Walnut Septum Membrane

**DOI:** 10.3390/molecules26216418

**Published:** 2021-10-24

**Authors:** Natasa P. Kalogiouri, Victoria F. Samanidou

**Affiliations:** Laboratory of Analytical Chemistry, Department of Chemistry, Aristotle University of Thessaloniki, 54124 Thessaloniki, Greece; kalogiourin@chem.auth.gr

**Keywords:** walnut septum, UAE, SPE, flavonoids, functional, HPLC-DAD

## Abstract

Walnut byproducts have been shown to exert functional properties, but the literature on their bioactive content is still scarce. Among walnut byproducts, walnut septum is a dry ligneous diaphragm tissue that divides the two halves of the kernel, exhibiting nutritional and medicinal properties. These functional properties are owing to its flavonoid content, and in order to explore the flavonoid fraction, an ultrasound-assisted (UAE) protocol was combined with solid phase extraction (SPE) and coupled to high-performance liquid chromatography with diode array detection (HPLC-DAD) for the determination of flavonoids in Greek walnut septa membranes belonging to Chandler, Vina, and Franquette varieties. The proposed UAE-SPE-HPLC-DAD method was validated and the relative standard deviations (RSD%) of the within-day and between-day assays were lower than 6.2 and 8.5, respectively, showing good precision, and high accuracy ranging from 90.8 (apigenin) to 97.5% (catechin) for within-day assay, and from 88.5 (myricetin) to 96.2% (catechin) for between-day assay. Overall, seven flavonoids were determined (catechin, rutin, myricetin, luteolin, quercetin, apigenin, and kaempferol) suggesting that the walnut septum is a rich source of bioactive constituents. The quantification results were further processed using ANOVA analysis to examine if there are statistically significant differences between the concentration of each flavonoid and the variety of the walnut septum.

## 1. Introduction

The development of analytical methodologies to characterize natural ingredients and produce high added value food products is a field of research that has attracted the interest of the scientific community, industry, and consumers, as well. During the last decades, there has been an increasing demand for foods that not only have high nutritional and sensorial quality but also deliver health promoting benefits through certain ingredients, namely “bioactive” or “functional” [1]. Moreover, emphasis is given on the valorization of food byproducts that combine nutritional value, flavor, and health benefits, as well. Towards this direction and in order to protect both public health and environment agri-food byproducts, generated in large amounts worldwide, can be exploited as a promising source of valuable compounds for novel food applications.

Walnuts (*Juglans regia* L.) are a valuable nutritional source with pleasant taste, consumed on a global scale. Walnut has been cultivated since 1000 BC, and it has naturally diverged to several cultivars, such as Chander, Vina, Franquette, Mellanaise, Lara, Marbot, Hartley Mayette, Serr, Tulare, Sorento, etc. [2]. Apart from the nutritional benefits, walnuts exert health-promoting properties, such as antioxidant, anti-inflammatory, antimicrobial, antidiabetic, hepatoprotective, anticancer, and cardioprotective, among others [3,4]. These benefits are owing to the presence of phytonutrients, and specifically to the phenolic compounds [5,6,7]. Even though the nutritional importance of the walnut is mainly related to the kernel, walnut byproducts, such as walnut husks, leaves, etc. [8,9], have been shown to exert functional properties, as well. Among walnut byproducts, walnut septum is a dry ligneous diaphragm tissue that divides the two halves of the kernel. Traditionally, the walnut septum has been used as a nutraceutical and medicinal material, and has been documented in the Chinese Pharmacopoeia [10]. The septum contains bioactive constituents, and it has been shown to exhibit antitumor, antioxidant, and immunoenhancement effects in vitro [11,12]. Phenolic compounds, and particularly flavonoids, are an important class of bioactive constituents that might be related to the functional properties of the walnut septum. Despite the fact that flavonoids are responsible for several pharmacological activities [13], the literature on the flavonoid content of walnut septum is still scarce.

Thus, the phenolic fraction of this unexplored byproduct has to be assessed with analytical methodologies. The generic analytical procedure for the determination of flavonoids in agricultural products involves first sample preparation, and then separation, detection, and quantification. Several traditional extraction techniques have been proposed for the extraction of flavonoids from natural products, including percolation, maceration, hydro-distillation, boiling, reflux, Soxhlet [14,15]. These techniques, however, present several disadvantages such as a lot of labor and time, large amounts of organic solvents, low selectivity, low extraction yield, and high cost [16]. For this reason, advanced green microextraction techniques are continually being developed to overcome these limitations. Green extraction techniques involve ultrasound-assisted extraction (UAE), microwave assisted extraction (MAE), supercritical fluid extraction (SFE), and solid phase extraction (SPE), among others. The current state-of-the art on the green extraction of natural constituents from food products has already been critically reviewed [4,17].

The main analytical technique used for the separation and determination of phenolic compounds is traditionally liquid chromatography (LC) coupled to UV, diode array detection (DAD), or mass spectrometric detectors (MS) [18,19,20,21]. The recent trends in analytical determination also involve the development of high-resolution mass spectrometric (HRMS) techniques, which provide separation efficiency and high accuracy in identifications, as it has already been reviewed [4,15,22,23]. Among these techniques, HPLC-DAD is a rapid analytical technique that enables the separation, identification, and quantification of flavonoids, offering several advantages in terms of sensitivity, specificity, ruggedness, and low cost of analysis. The accurate determination of the flavonoids and the further quantification of such analytes that exist in trace levels in food matrices is a challenging task. The further processing of the quantification results with statistical tools enhances the conclusions derived from the experimental data, allowing the discovery of trends and behaviors among the samples.

In this work, a green UAE-SPE-HPLC-DAD methodology was developed and validated for the determination of flavonoids in walnut septum belonging to different varieties (Chandler, Vina, and Franquette) cultivated in Greece. The determined analytes were quantified, and the results were further analyzed by one-way ANOVA to examine if there are statistically significant differences between the analytes’ concentrations and the walnut variety. To the best of our knowledge, this is the first report of assessing the flavonoid profile of Greek walnut septa.

## 2. Results and Discussion

### 2.1. Method Development and Validation

An HPLC-DAD methodology was developed and validated to assess the flavonoid profile of walnut septum and all the analytical parameters, including the calibration curves, linear range, the determined coefficients (r^2^), accuracy and precision, limits of detection (LODs), and limits of quantification (LOQs) are presented in Table 1. The analytical curves presented an adequate fit when submitted to the lack-of-fit test (F_calculated_ was less than F_tabulated_ in all cases), with r^2^ above 0.99, proving that they can be used for the quantification of the flavonoids. The LOQs were found to range between 0.30 μg/g and 0.90 μg/g, while the LODs were calculated over the range 0.10 μg/g to 0.30 μg/g. The RSD% of the within-day (*n* = 6) and between-day assays (*n* = 3 × 3) were lower than 6.2, and 8.5, respectively, showing adequate precision. The accuracy was assessed by means of relative percentage of recovery (%R) at low, medium, and maximum concentration levels of 1, 5, and 10 μg/g, and the results were acceptable, ranging from 90.8 (apigenin, at 10 μg/g concentration level) to 97.5% (catechin, at 10 μg/g concentration level) for within-day assay (*n* = 6) (Table 2), and from 88.5 (myricetin, at 1 μg/g concentration level) to 96.2% (catechin, at 5 μg/g concentration level) for between-day assay (*n* = 3 × 3) (Table 3).

### 2.2. Walnut Septum Analysis

**Figure 1:**
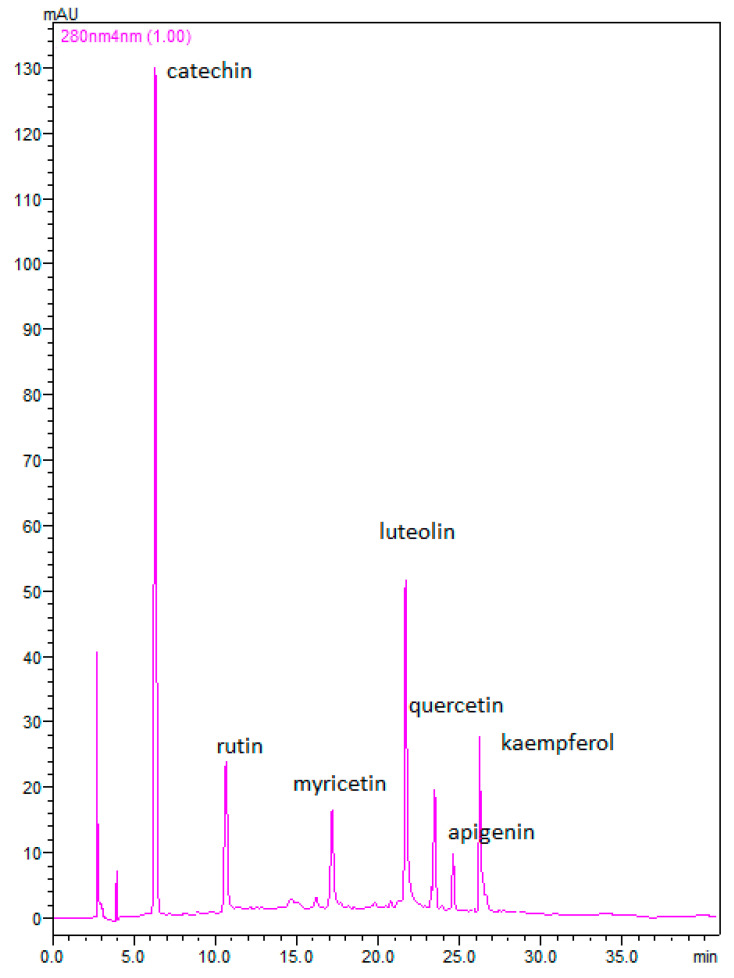
Characteristic chromatogram of a walnut septum extract; monitored at 280 nm.

Twenty-four walnut septum membranes belonging to the varieties Chandler, Vina, and Franquette were analyzed in triplicate and the flavonoids: catechin, rutin, myricetin, luteolin, quercetin, apigenin, and kaempferol were determined. The chromatographic identification results, including the retention times (R_ts_) of the analytes, and their respective maximum absorption wavelengths (*λ*, nm) are presented in Table 4. Figure 1 illustrates the chromatographic separation of the flavonoids in a walnut septum extract that was monitored at 280 nm.

### 2.3. Quantitative Analysis of Flavonoids

The walnut septa were analyzed in triplicate (*n* = 3). The identified flavonoids (catechin, rutin, myricetin, luteolin, quercetin, apigenin, and kaempferol) were quantified on the basis of their maximum absorption wavelengths. The presence of the determined flavonoids was linked to certain positive health effects and other bioactive functions that have already been reported in the literature to highlight the potential functional activity of the analyzed byproduct [4]. Table 5 presents the quantification ranges and mean values (±SD) of the determined flavonoids in the walnut septa belonging to Chandler, Vina, and Franquette variety. Box and Whisker plots were constructed to graphically illustrate the concentrations of the flavonoids and present the distributional characteristics of each variety (Chandler, Vina, Franquette). The quantification results were further analyzed with ANOVA to examine if there are statistically significant differences between the concentrations of the determined flavonoids and the varieties of the walnut septa.

#### 2.3.1. Catechin

**Figure 2:**
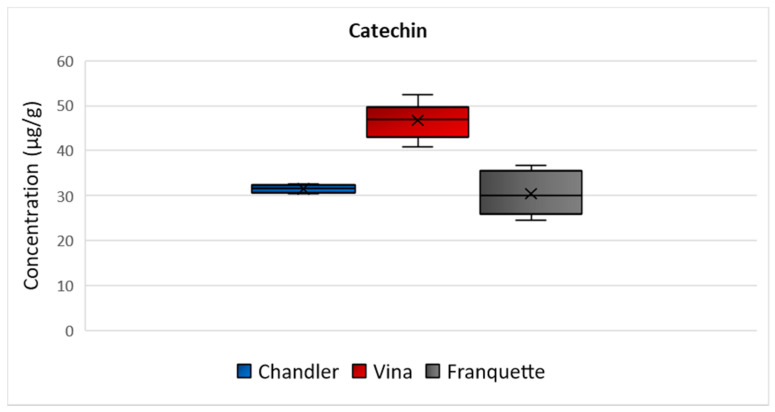
Box and Whisker plot for the concentration of catechin among walnut septa, belonging to the Chandler, Vina, and Franquette varieties.

Catechin was the flavonoid detected in higher concentration in all the analyzed septa. Specifically, the highest mean concentration was determined in Vina septa (47 μg/g), and then Chandler (32 μg/g), and Franquette (31 μg/g) varieties followed (Figure 2). The ANOVA analysis exhibited a statistically significant difference in catechin concentration among all of the walnut septa of the different varieties with *p* = 0.04. The concentration ranges indicate that the walnut septum is rich in catechin, and it could be effectively used as a food additive to increase the antioxidant potential of food products, enrich feeds, while it could also be used as a functional ingredient of novel functional food products, including soft beverages, teas, infusions, etc. Several health properties have been associated with catechins, as they have proven to be promising protective agents against diabetes, arterial hypertension and ischemic stroke, obesity, metabolic syndrome [24]. Moreover, catechins demonstrate significant biological activities against oral cancer, breast cancer, Alzheimer’s disease, Parkinson’s disease [24]. Furthermore, catechin displays unique features responsible for several pharmacological and biological properties, as it possesses the ability to block ROS-induced chain reactions, acting as a promising antioxidant [25]. It also demonstrates significant antidiabetic function, via hepatoprotective, antineurodegenarative effects, insulin-mimetic effects, hindering amyloid formation [24].

#### 2.3.2. Rutin

**Figure 3:**
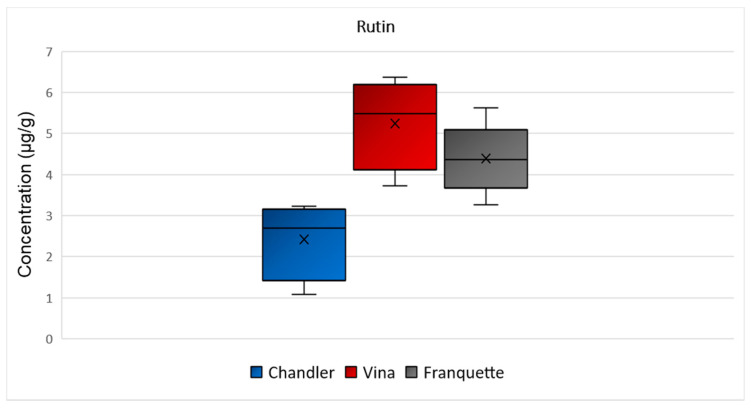
Box and Whisker plot for the concentration of rutin among walnut septa belonging to the Chandler, Vina, and Franquette varieties.

Rutin was determined over the range 1–3 μg/g in Chandler walnut septa, and over the ranges 4–6 μg/g and 3–6 μg/g in Vina and Franquette walnut septa, respectively (Figure 3). A statistically significant difference was observed for the concentration of rutin among the septa of the different varieties (*p* = 0.002). Rutin possesses promising antioxidant potential and exhibits significant biological properties, thus, playing an essential role in the human body’s numerous physiological functions [26]. According to the literature, rutin may provide a wide variety of therapeutic effects, such as antiallergic, antiviral, antihypertensive, vasoactive, cytoprotective, anti-inflammatory, antiprotozoal, hypolipidemic, antispasmodic, anticarcinogenic, antibacterial, and antiplatelet activities [27]. Furthermore, rutin contributes to the strengthening of the blood vessels capillaries, due to its high radical scavenging capacity, thereby preventing fragility-associated hemorrhagic disorders in humans [28].

#### 2.3.3. Myricetin

**Figure 4:**
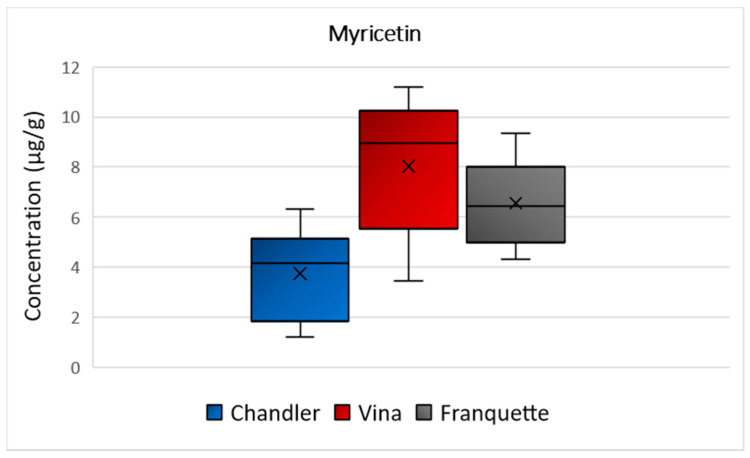
Box and Whisker plot for the concentration of myricetin among walnut septa belonging to the Chandler, Vina, and Franquette varieties.

The mean concentration values of myricetin were equal to 4 μg/g, 8 μg/g, and 7 μg/g in Chander, Vina, and Franquette walnut septa, respectively (Figure 4). Τhe ANOVA analysis showed that there is significant statistically difference among the septa of the different varieties (*p* = 0.002). Great scientific interest has been raised in myricetin, which has been shown to exhibit antioxidant, anti-inflammatory, antiviral, and anticarcinogen activities. It is functioning as an antineoplastic agent in human patients, since it has demonstrated strong suppressive effects on the activities of several types of cancer cells (e.g., cancer cell invasion or metastasis), thus, regulating apoptosis, and inhibitory properties on their proliferation [29,30,31,32].

#### 2.3.4. Luteolin

**Figure 5:**
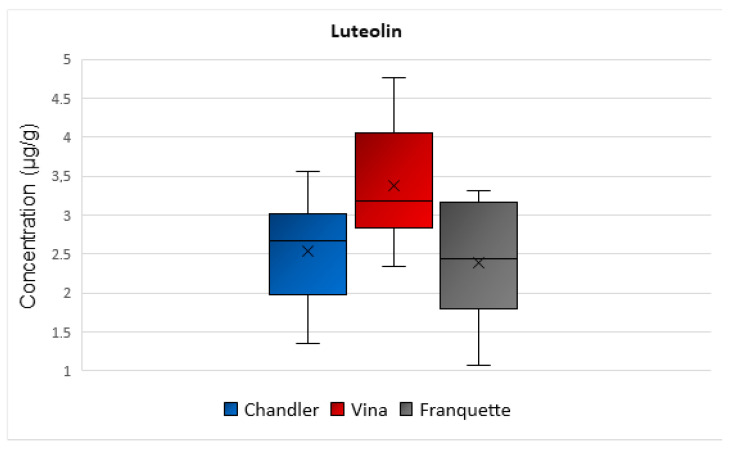
Box and Whisker plot for the concentration of luteolin among walnut septa belonging to the Chandler, Vina, and Franquette varieties.

The mean concentration values of luteolin were equal to 3 μg/g, 3 μg/g, and 2 μg/g in Chandler, Vina, and Franquette walnut septa, respectively (Figure 5). Statistically significant differences were observed among the luteolin concentrations determined in septa of the different varieties (*p* = 0.039). As for its biological action, luteolin is a naturally occurring flavonoid with a yellow crystalline appearance, and scientific research has indicated that it may display multiple cellular effects, hence, favorably affecting human health. It may exhibit antioxidant properties, protecting cells from ROS induced damage or act as an antineoplastic, anti-inflammatory, antimicrobial, or estrogenic regulatory compound and prevent liver diseases [33].

#### 2.3.5. Quercetin

**Figure 6:**
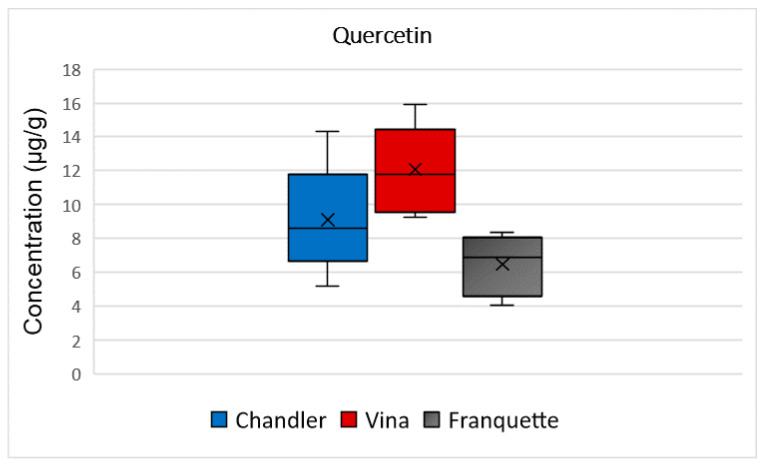
Box and Whisker plot for the concentration of quercetin among walnut septa belonging to the Chandler, Vina, and Franquette varieties.

Quercetin was determined at 9 μg/g, in Chandler walnut septa. The mean concentration of quercetin was higher in walnut septa belonging to the Vina variety (12 μg/g), and lower for those belonging to the Franquette variety (6 μg/g), as it is presented in Figure 6. The ANOVA analysis showed that there is statistically significant difference among the quercetin concentration and the analyzed varieties (*p* = 0.0008). According to the literature, quercetin is responsible for the bitter flavor of foods, and in synergy with myricetin and other phenolic compounds has been recently used as food additives to protect meat products against bacteria’s development [34]. It demonstrates several significant health-promoting functions, including cardioprotective, anti-ulcer, antidiabetic, antioxidant properties and chemopreventive potential [35]. It may also produce anti-inflammatory and anti-allergy effects through the inhibition of the lipoxygenase and cyclooxygenase pathways, thereby reducing the production of pro-inflammatory or pro-oxidant mediators [35].

#### 2.3.6. Apigenin

**Figure 7:**
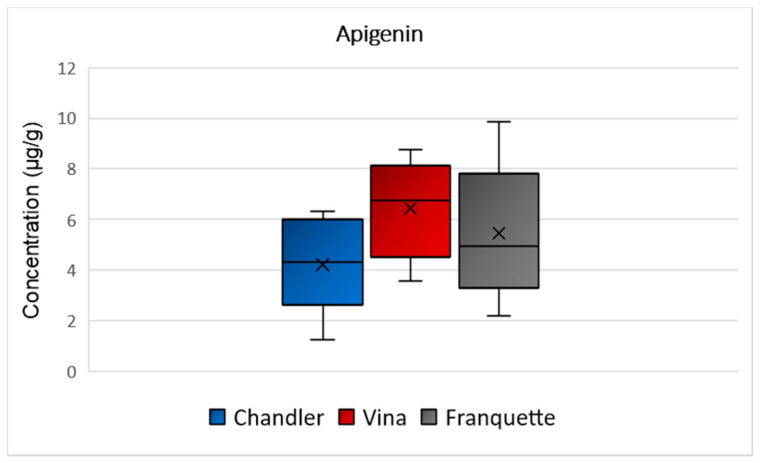
Box and Whisker plot for the concentration of apigenin among walnut septa belonging to the Chandler, Vina, and Franquette varieties.

Apigenin was determined at mean concentrations of 4 μg/g, 6 μg/g, and 5 μg/g in Chandler, Vina, and Franquette walnut septa, respectively (Figure 7). The ANOVA analysis showed that there was no statistically significant difference among the apigenin concentrations in the analyzed septa of the different varieties (*p* = 0.147). Traditionally, extracts, oils, and teas from plants with a high content of apigenin were used for its soothing qualities as a sedative, mild analgesic and sleep medication [36], reinforcing the idea that walnut septa could be used in infusion making. Moreover, in the food industry, apigenin possesses a role as a flavoring or adjuvant agent, enhancing the human body’s response to antigens [35]. It may yield antiproliferative and antimetastatic effects, suppressing the formation and inducing malignant tumor cells [28]. Furthermore, it could prevent skin or colon cancer and acts as an anti-inflammatory, antioxidant, antiallergic, antimicrobial, antiviral, cardioprotective, and neuroprotective agent [28].

#### 2.3.7. Kaempferol

**Figure 8:**
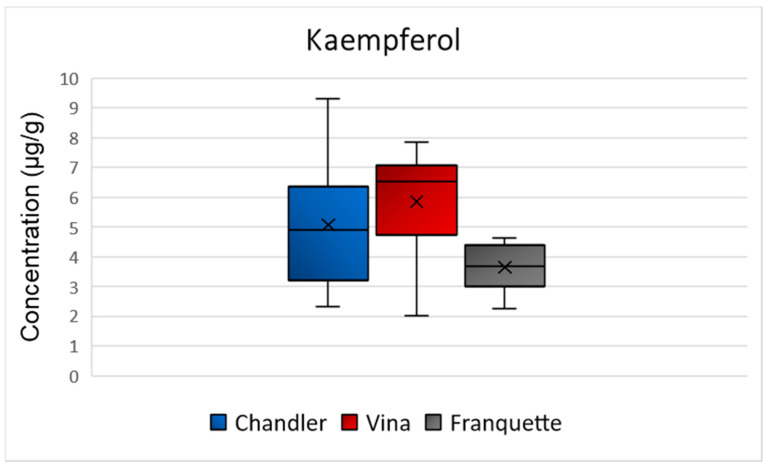
Box and Whisker plot for the concentration of kaempferol among walnut septa belonging to the Chandler, Vina, and Franquette varieties.

The highest mean concentrations of kaempferol were observed in the Vina variety (5 μg/g), while Chandler variety followed with 6 μg/g, and Franquette exhibited the lowest mean concentration of 4 μg/g (Figure 8). No statistically significant difference was observed for the concentration of kaempferol among the septa of the different varieties (*p* = 0.06). As for its biological action, kaempferol is a natural dietary flavonoid, potentially acting as chemopreventive agent [37], protecting against oxidative stress and inflammatory chronic disorders.

### 2.4. Effect of the Variety on the Phenolic Content

The determination of the seven flavonoids clearly indicates that walnut septum is a byproduct that is rich in flavonoids, and, thus, could be characterized as a “nutraceutical” raw ingredient. This is also supported by the quantification ranges of each individual flavonoid identified. This fruit septum of walnuts could be utilized as a substance for medicinal purposes, while it could be also further exploited for the development of novel functional foods and beverages, and for the enrichment of feeds, as well.

**Figure 9:**
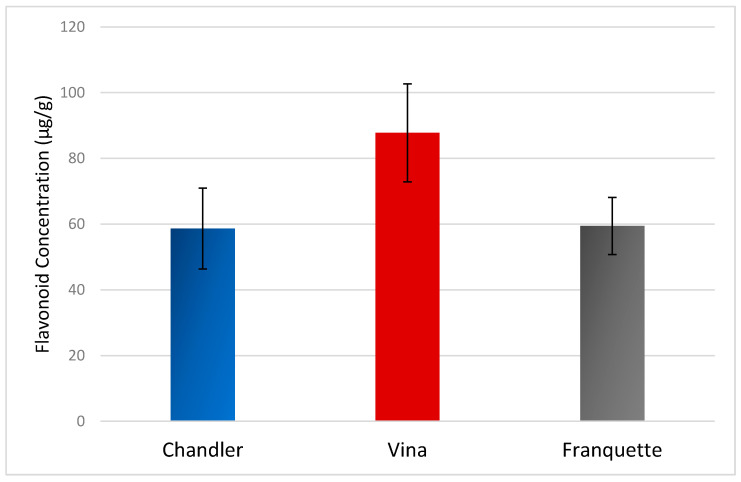
Average flavonoid concentration of walnut septum belonging to Chandler, Vina, and Franquette varieties.

Except for the functional properties that have been attributed to each individual analyte, and were discussed in detail in Section 2.3, the findings of this research also demonstrated that the flavonoid content of the walnut septum is affected by the variety. The sum of the individually quantified flavonoids showed that the walnut septum of the Vina variety exhibited the highest flavonoid content (88 ± 15 μg/g). The flavonoid contents of the septa belonging to Chandler variety, and the Franquette variety were approximately similar, with calculated concentrations of 59 ± 12 μg/g, and 60 ± 9 μg/g, respectively. The graph in Figure 9 presents the sum of the mean values of the individual concentrations of the flavonoids determined in the walnut septa belonging to Chandler, Vina, and Franquette varieties. Considering that the analyzed samples originated from the same geographical origin (Thessaly, Greece), the variation in the concentration of the flavonoids could be linked to the genetic fingerprint of each variety.

Furthermore, the ANOVA analysis showed that there is statistically significant difference between the concentrations of catechin, rutin, myricetin, luteolin, quercetin, and the walnut variety. Furthermore, PCA analysis was employed to investigate the similarities in the flavonoid content of the septa according to their variety, and the first two PCs explained the 70% of variance. The PCA score plot and loading plot are presented in the Supplementary Material in Figures S1 and S2, respectively. Even though several works have already associated the bioactive content of the walnut kernel with the geographical origin of walnuts [18,38], and the variety [39,40], these are the first reports relating the flavonoid content of the walnut septum with the variety. 

## 3. Materials and Methods

### 3.1. Chemicals and Reagents

Methanol (MeOH) and acetonitrile (ACN), HPLC grade, were acquired from Merck (Darmstadt, Germany). Acetic acid 99% and trifluoroacetic acid (TFA) 99% were purchased from Sigma-Aldrich (Steinheim, Germany). The LiChrolut RP-18 (C18, 3 mL, 500 mg) SPE cartridges used were supplied by Merck (Darmstadt, Germany). Ultrapure water was provided by a Milli-Q^®^ purification system (Millipore, Bedford, MA, USA). The flavonoids catechin 98%, rutin 98%, myricetin 98%, luteolin 98%, quercetin 98%, kaempferol 98%, and apigenin 98% were supplied by Sigma-Aldrich (Steinheim, Germany). Stock standard solutions at 1000 mg/L concentration level were prepared and stored in dark brown glass bottles at −20 °C. Working standard solutions were prepared in MeOH after appropriate dilution of the stock solutions every laboratory day, before analysis.

### 3.2. Instrumentation

A quaternary low-pressure gradient HPLC–DAD system by Shimadzu (Kyoto, Japan) was used for analysis. The HPLC system consisted of: (a) an FCV-10ALVP mixing system, (b) a Rheodyne 7725i injection valve (Rheodyne, Cotati, CA, USA), and a 20 µL loop for sample injection, (c) an LC- 10ADVP pump equipped with a Shimadzu SCL-10ALVP System Controller, (d) an SPD-M10AVP photodiode array detector. Real time analysis monitoring and post run processing were carried out using the software Lab Solutions-LC solutions, supplied by Shimadzu. A glass vacuum filtration apparatus, acquired by Alltech Associates (Deereld, IL, USA), and nylon 0.2 µm membrane Filters (Alltech Associates, Chicago, IL, USA) were utilized for the filtration of the mobile phase, and a DGU-10B de-gassing unit with helium was used for degassing. A vortexer purchased from FALC Instruments (Treviglio (BG), Italy) was used for sample agitation. Centrifugation was carried out using a HermLe centrifuge, model Z-230 (B. HermLe, Gosheim, Germany). An ultrasonic bath (MRC: DC-150H) by MRC (Essex, UK) was used for sample preparation. For evaporation, after SPE extraction, a ReactiVap 9-port evaporator model 18,780 by Pierce (Rockford, IL, USA) was utilized. For sample filtration, prior to the injection in the chromatographic system, Q-Max RR syringe filters (0.45 µm nylon membrane) were purchased from Frisenette ApS (Knebel, Denmark).

### 3.3. Chromatographic Separation and Analysis

The chromatographic separation of the flavonoids was achieved on a C18 UniverSil column (250 mm × 4.6 mm, 5 µm), supplied by Fortis Technologies Ltd. (Neston, UK). A reverse-phase HPLC assay was carried out using a gradient system with 1 mL/min flow rate, thermostated at 30 °C. The mobile phase consisted of (A) 1% acetic acid in water, and (B) ACN. The gradient elution program begun with 80:20, *v/v* (A: B), gradually increasing to 50:50, *v/v* (A: B), in the following 25 min, and then remaining constant for the next 5 min. Τhe initial conditions were restored for 10 min, prior to the next injection. The injection volume was 20 μL of solution and the total run time was less than 25 min for each injection. For peak identification, the R_ts_ of the peaks of the real samples were compared with the R_ts_ of the standard compounds, along with the spectral information provided by the DAD detector that operated over the range 280–400 nm. Peak monitoring and quantitation were performed at the maximum wavelength of each analyte.

### 3.4. Sample Collection

Twenty-four walnut septa samples were created after crushing walnuts in the laboratory using a wooden hammer, and carefully removing the walnut septa. Each walnut septum sample was a bulk sample that consisted of ten walnut septa originating from the same tree. In this way, eight bulk walnut septum samples were created in the laboratory for each variety (Chandler, Vina, Franquette). All the walnut samples were collected during the harvesting period of November 2020 from Kokkinogi, in Thessaly, Greece (40°2′2.62″ N 22°9′54.00″ E).

### 3.5. Sample Preparation

The samples were homogenized in a porcelain mortar and stored at −20 °C until analysis. For sample preparation, 50 mg of each homogenized bulk sample was weighted in 2-mL Eppendorf tubes, and then, 0.5 mL of 0.05% TFA in methanol: water at 60:40 ratio (*v/v*), was added [18]. The mixture was vortexed for 1 min and, then ultra-sound assisted extraction took place in an ultrasonic bath for 10 min at 25 °C. Each sample was centrifuged for 10 min at 10,000 rpm and, then, the supernatant was collected, according to Kalogiouri et al. [18]. The extract was further diluted with water at a final volume of 2 mL. The diluent was purified using a modified version of the SPE protocol proposed by Bajkacz et al. [41]. The LiChrolut RP-18 (C18, 3 mL, 500 mg) SPE cartridges were used for this purpose. First, the C18 column was conditioned with 2 mL MeOH, followed by 2 mL of water. Then, the diluted sample extract was passed through the sorbent at a flow rate of approximately 1 mL/min. The analytes were eluted with 3 mL MeOH and the eluates were evaporated to dryness with nitrogen. The residues were dissolved in 1 mL MeOH, and filtered through 0.22 μm nylon syringe filters. Finally, 20 μL was injected into the chromatographic system.

### 3.6. Method validation and Quantification

Linearity, selectivity, LODs and LOQs, within-day, and between-day accuracy and precision were evaluated. Linearity was examined by testing the lack-of-fit of the calibration calibration curves over the range 1–10 μg/g. The slopes, intercepts, and the determination coefficients of each analyte were calculated using last square linear regression analysis. LODs and LOQs were calculated by the equations:

LOD = 3.3 × *Sa*/*b* (1)

LOQ = 10 × *Sa*/*b* (2)

where, *Sa* is the standard error of the intercept α; and b is the slope of the calibration curve [42]. Accuracy was evaluated after spiking a bulk sample at 1, 5, and 10 mg/kg concentration level, and analysis was performed in triplicate. Accuracy was expressed as relative recovery, and precision was expressed as relative standard deviation (RSD%). Repeatability, expressed as within-day precision, was assessed in six replicates (*n* = 6), and reproducibility, expressed as between-days precision, was assessed after analyzing the spiked bulk samples within three consecutive days (*n* = 3 × 3). The analytes were quantified using the corresponding calibration curves of the standards. For the quantification of the analytes with high concentrations that exceeded beyond the linear range, such as catechin, the extracts were further diluted with MeOH and re-injected in the chromatographic system to ensure that their calculated concentration was within the linear range of the curves.

### 3.7. Chemometric Analysis

The quantification results were processed with one-way analysis of variance (ANOVA), using the data analysis tool of Microsoft Excel (Microsoft, Redmond, WA, USA). ANOVA was applied to examine potential statistically significant differences among the flavonoids’ concentrations and the different varieties of the walnut septa (Chandler, Vina, Franquette). A *p*-value was used for a confidence level of 95% to evaluate the results. If the *p*-value was calculated higher than 0.05, there was no statistically significant difference, and in the cases that the *p*-value was lower than 0.05, there was statistically significant difference among the samples. Principal Component Analysis (PCA) was employed to investigate the interrelationships among the determined flavonoids and the samples belonging to the different varieties (Chandler, Vina, and Franquette). PCA was created in R using the MetaboAnalystR package [43].

## 4. Conclusions

A UAE-SPE-HPLC-DAD analytical method was developed and validated to determine flavonoids in twenty-four walnut septa belonging to Chandler, Vina, and Franquette varieties cultivated in Greece. Overall, seven flavonoids were determined (catechin, rutin, myricetin, luteolin, quercetin, apigenin, and kaempferol), indicating that the walnut septum is rich in flavonoids, and it could be further exploited and utilized in the pharmaceutical industry, and in the food and feed industry, as well. The calculated concentrations of all the individual flavonoids were further analyzed with ANOVA, and the results indicated that there is statistically significant difference among the concentrations of catechin, rutin, myricetin, luteolin, quercetin, and the variety of the analyzed septa, demonstrating that the flavonoid content of the walnut byproduct is affected by the variety. PCA analysis was employed to investigate the similarities in the flavonoid content of the septa according to their variety, and the first two PCs explained the 70% of variance. Overall, the findings of this work suggest that the walnut septum is a promising raw ingredient with functional properties and several potential uses.

## Figures and Tables

**Table 1:** HPLC-DAD method analytical parameters.

Compound	Calibration Equation y = (a ± Sa) + (b ± Sb)x (Linear Range: 1–10 μg/g)	r^2^	F_calc_	F_tab_	LOD (μg/g)	LOQ (μg/g)
catechin	y = (1095 ± 1115) + (11808 ± 305)x	0.997	7.9 × 10^−9^	0.2334	0.31	0.94
rutin	y = (389 ± 1200) + (19857± 204)x	0.995	1.9 × 10^−9^	0.2334	0.20	0.60
myricetin	y = (989 ± 1450) + (20005 ± 424)x	0.993	5.6 × 10^−9^	0.2334	0.24	0.72
luteolin	y = (1017 ± 1608) + (17008 ± 440)x	0.995	2.9 × 10^−9^	0.2334	0.20	0.60
quercetin	y = (−1032 ± 1128) + (18404 ± 153)x	0.993	6.5 × 10^−9^	0.2334	0.20	0.60
apigenin	y = (1732 ± 152) + (1745 ± 665)x	0.994	4.6 × 10^−7^	0.2334	0.29	0.87
kaempferol	y = (1710 ± 54.3) + (19045 ± 685)x	0.996	1.7 × 10^−9^	0.2334	0.29	0.90

Ftab: Ftabulated, Fcalc: Fcalculated, LOD: limit of detection, LOQ: limit of quantitation.

**Table 2:** %Recoveries (%R, *n* = 6) for the evaluation of repeatability.

Compound	%R Low Conc. Level (1 μg/g)	%RSD	%R Medium Conc. Level (5 μg/g)	%RSD	%R Maximum Conc. Level (10 μg/g)	%RSD
catechin	97.1	5.3	96.4	6.2	97.5	5.3
rutin	93.5	5.1	92.5	4.5	98.4	2.5
myricetin	91.8	4.3	94.4	5.2	91.2	1.7
luteolin	94.2	3.8	95.6	4.6	93.6	3.9
quercetin	93.6	3.4	98.8	4.2	94.1	4.2
apigenin	94.4	5.4	91.7	6.1	90.8	5.1
kaempferol	92.1	2.8	93.5	3.2	93.5	2.9

Conc.: Concentration.

**Table 3:** %Recoveries (%R, *n* = 3 × 3) for the evaluation of intermediate precision.

Compound	%R Low Conc. Level (1 μg/g)	%RSD	%R Medium Conc. Level (5 μg/g)	%RSD	%R Maximum Conc. Level (10 μg/g)	%RSD
catechin	95.1	5.2	96.2	4.8	92.1	6.4
rutin	93.5	7.1	95.7	5.9	95.2	5.2
myricetin	88.5	6.2	93.4	6.5	94.4	6.1
luteolin	91.8	5.8	89.2	7.5	93.5	8.5
quercetin	94.5	7.8	90.3	8.1	92.3	6.9
apigenin	93.7	6.3	91.1	7.4	94.2	5.4
kaempferol	95.5	5.8	88.9	6.6	95.4	7.3

Conc.: Concentration.

**Table 4:** Retention time and maximum absorption wavelength of the determined flavonoids.

Compound	Chemical Structure	Rt	*λ* (nm)
catechin	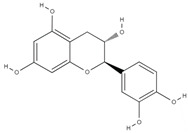	5.8	278
rutin	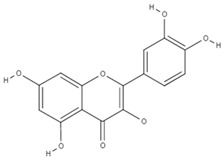	10.1	353
myricetin	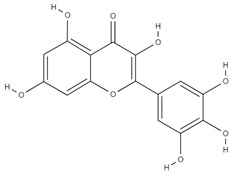	16.5	370
luteolin	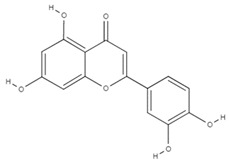	21.1	356
quercetin	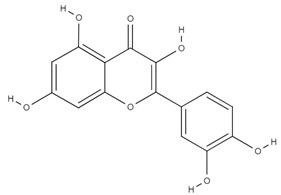	22.7	378
apigenin	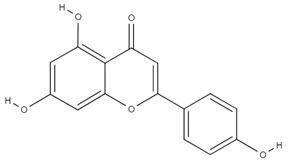	24.5	360
kaempferol	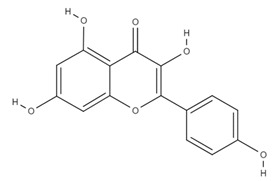	26.1	360

Rt: retention time.

**Table 5:** Quantification results of the flavonoids determined in walnuts septa belong to Chandler, Vina, and Franquette varieties (samples analyzed in triplicate, *n* = 3).

Variety	Chandler		Vina		Franquette	
Compound	Concentration Range (μg/g)	Mean Value (μg/g ± SD)	Concentration Range (μg/g)	Mean Value (μg/g ± SD)	Concentration Range (μg/g)	Mean Value (μg/g ± SD)
catechin	30–33	32 ± 5	41–53	47 ± 3	25–37	31 ± 1
rutin	1–3	2.4 ± 0.2	3–6	5 ± 2	3–6	4 ± 2
myricetin	1–6	3.8 ± 0.8	3–11	8 ± 2	5–9	7 ± 2
luteolin	1–4	2.6 ± 0.2	2–5	3.4 ± 0.9	1–3	2.4 ± 0.3
quercetin	5–14	9 ± 1	9–16	12 ± 4	4–8	6 ± 2
apigenin	1–6	4 ± 1	4–9	6 ± 2	2–10	5.5 ± 0.7
kaempferol	2–9	5 ± 3	2–8	6 ± 1	2–5	3.7 ± 0.9

## Supplementary Materials

The following are available online. Figure S1: PCA 3D score plot in color presenting pairwise correlation between PCs in the clustering between walnut septa belonging to Chandler, Vina, and Franquette variety; Figure S2: PCA loading plot showing the projection of the data set in PC1 × PC2 plane.

## Sample Availability

Samples of the compounds are available from the authors.
