# Supplementary material for: A Validated Ultrasound-Assisted Extraction Coupled with SPE-HPLC-DAD for the Determination of Flavonoids in By-Products of Plant Origin: An Application Study for the Valorization of the Walnut Septum Membrane
Source: Molecules. 2021 Oct 24;26(21):6418. doi: 10.3390/molecules26216418 (PMC8588283; doi:10.3390/molecules26216418)
Supplement: Supplementary file 1 [file molecules-26-06418-s001.zip › molecules-1438160-supplementary.pdf]

Supplementary Material

A validated Ultrasound-Assisted Extraction coupled with SPE-HPLC-DAD for the Determination of Flavonoids in by-Products of Plant Origin: An Application Study for the Valorization of the Walnut Septum Membrane

Natasa P. Kalogiouri and Victoria F. Samanidou

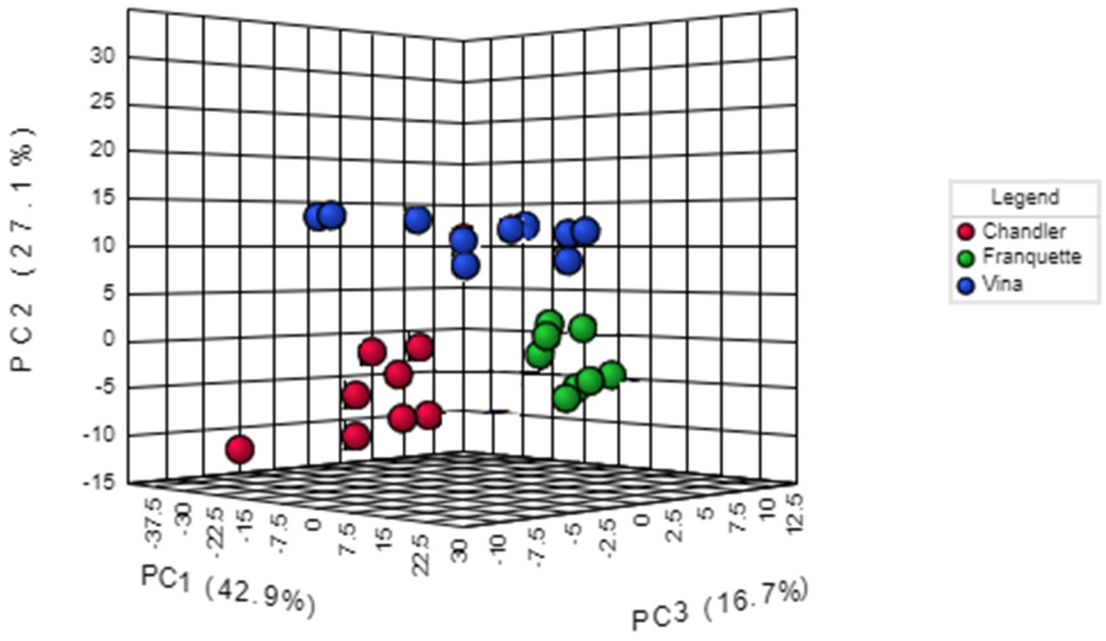

Figure S1. PCA 3D score plot in color presenting pairwise correlation between PCs in the clustering between walnut septa belonging to Chandler, Vina, and Franquette variety.

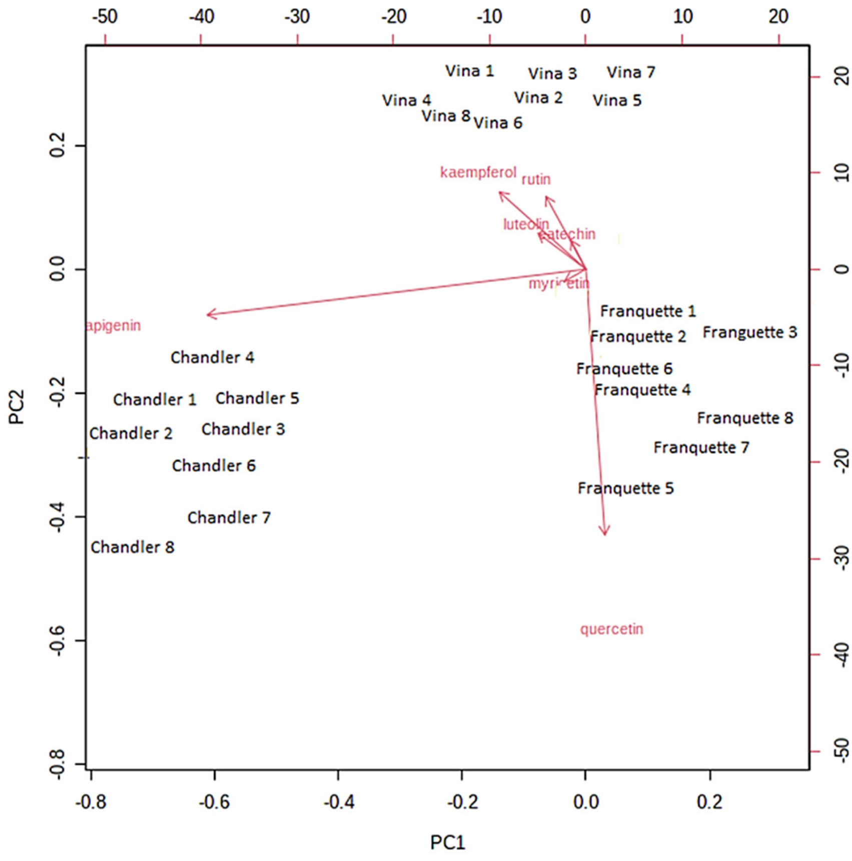

Figure S2. PCA loading plot showing the projection of the data set in PC1 x PC2 plane.
